# Ultrahigh-Performance Supercritical Fluid Chromatography–Multimodal Ionization–Tandem Mass Spectrometry as a Universal Tool for the Analysis of Small Molecules in Complex Plant Extracts

**DOI:** 10.1021/acs.analchem.3c03599

**Published:** 2024-02-01

**Authors:** Kateřina Plachká, Veronika Pilařová, Štefan Kosturko, Jan Škop, Frantisek Svec, Lucie Nováková

**Affiliations:** Department of Analytical Chemistry, Faculty of Pharmacy in Hradec Králové, Charles University, Heyrovského 1203, 500 05 Hradec Králové, Czech Republic

## Abstract

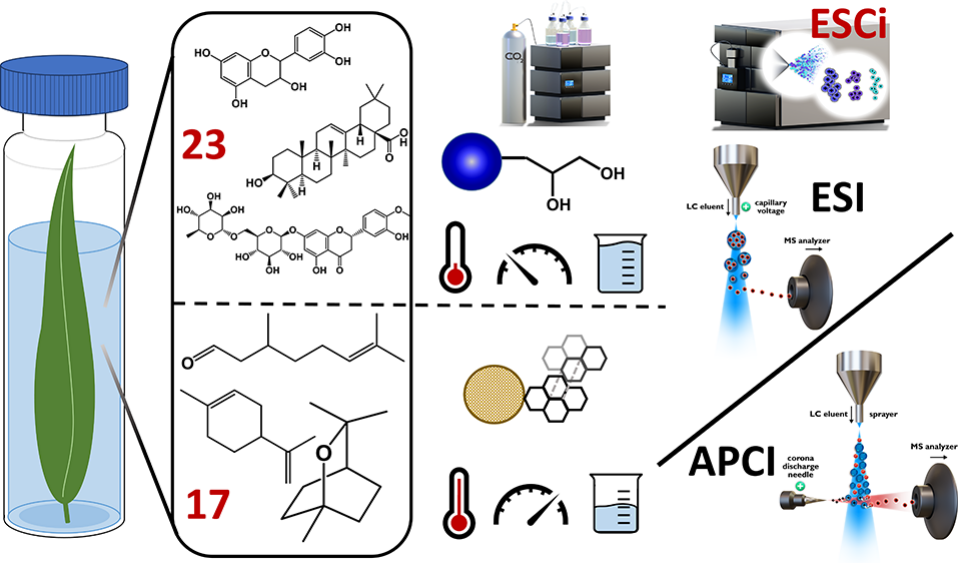

Complex analysis of plant extracts usually requires a combination of several analytical approaches. Therefore, in this study, we developed a holistic two-injection approach for plant extract analysis, which is carried out within one instrument without the need for any manual intervention during the analysis. Ultrahigh-performance supercritical fluid chromatography (UHPSFC) was employed for the analysis of 17 volatile terpenes on a porous graphitic carbon column within 7.5 min, followed by analysis on short diol column where flavonoids, phenolic acids, and terpenoic acids were analyzed within 15.5 min. A multimodal ionization source combining electrospray and atmospheric pressure chemical ionization (ESCi) was selected for mass spectrometry detection as a simultaneous ionization of both lipophilic and polar compounds was required. The quantitative aspects of the final UHPSFC-ESI/ESCi-MS/MS two-injection approach were determined, and it was applied to the analysis of *Eucalyptus sp.* extracts prepared by supercritical fluid extraction. Current methods reported in the literature typically require a labor-intensive combination of liquid and gas chromatography for the complex analysis of plant extracts. We present for the first time a new UHPSFC approach requiring only a single instrument that provides an alternative approach to the analysis of complex plant extracts.

The analysis of plant extracts is often challenging. Indeed, more than 200,000 primary and secondary metabolites have been identified in the plant kingdom, varying in their content, structure, and physicochemical properties from small nonpolar to very polar large molecules.^1^ Thus, different analytical techniques are required and used for the analysis of different groups of metabolites, such as terpenes, flavonoids, alkaloids, carotenoids, and lipids, with emphasis on efficiency, selectivity, and sensitivity.^1,2^ Nonpolar molecules, including terpenes and other volatiles, are mostly separated using gas chromatography (GC), which can also be a suitable choice for the analysis of nonvolatile compounds after derivatization. On the other hand, liquid chromatography (LC) is widely used in the analysis of various compounds due to the availability of large number of stationary phases and chromatographic modes.^2,3^

In recent years, ultrahigh-performance supercritical fluid chromatography (UHPSFC) has established a strong position among the separation techniques in various application fields.^4,5^ The unique physicochemical properties of the mobile phase consisting of carbon dioxide, organic modifier, and additive, allow the separation of nonpolar, medium polar to polar compounds.^1,6^ Due to the many possibilities of method tuning, including not only mobile phase composition and stationary phase selection but also pressure, temperature, and flow rate adjustment, the continuous change of conditions from SFC to LC is possible. In the early 1990s, the first attempts to separate medium polar compounds were made when using polar packed columns in SFC^7^ and/or unified chromatography (UC) as a concept where the single chromatographic system could carry out analysis in different modes, including all GC, LC, and SFC.^8^ Indeed, the current UC combining SFC and LC mode of separation has been successfully applied in the analysis of natural dyes^9,10^ and short-chain bioactive peptides^11^ using a mobile phase gradient from supercritical CO_2_ to neat organic solvent together with pressure and flow rate gradients. In addition, Losacco et al. introduced dual-gradient UC combining two gradients: the gradient of modifier in CO_2_ and the gradient of water in the organic modifier.^12^ A similar approach called UC-HILIC (unified chromatography-hydrophilic interaction liquid chromatography) was published as a proof of concept for metabolomic analysis by Si-Hung in 2023.^13^ However, none of these approaches focused on the analysis of both nonpolar small compounds, mainly analyzed by GC, and polar compounds within one run.

A hyphenation of SFC with mass spectrometry (MS) has been necessary to increase the selectivity and sensitivity of the methods, as SFC is a promising tool in many application fields. Various interfaces have been adopted over the years.^4,14,15^ Similar to LC-MS, the atmospheric pressure ionization techniques are typically used, with electrospray ionization (ESI) as the dominant technique suitable for compounds with moderate to high polarity.^14^ Atmospheric pressure chemical ionization (APCI) and atmospheric pressure photoionization source (APPI) are preferred for nonpolar to moderately polar compounds such as fat-soluble vitamins,^16^ steroids,^17^ triterpenoids,^18^ apocarotenoids,^19^ and oily samples.^20,21^ SFC-APCI-MS method was developed for the determination of 23 volatile compounds using a newly developed poly(styrene-*co*-divinylbenzene) column that has not been introduced to the market yet.^22^*Citrus limon* essential oil was analyzed by SFC-APPI-MS using a porous graphitic carbon column.^23^ Only few of the terpenes such as limonene, pinene, citral, and menthol targeted in our study were analyzed also within these two studies.^22,23^ Moreover, a complex analysis of plant extracts also requires the analysis of more polar compounds, such as flavonoids and phenolic acids, which can be challenging to ionize in APCI and APPI. A multimodal ion source combining APCI and ESI has been introduced by several vendors, such as Agilent, ThermoFisher, and Waters. This multimodal ion source allows quick switching between ESI and APCI improving the ionization of nonpolar compounds while preserving ESI responses for more polar analytes. However, no application to real-life samples taking advantage of this universal ionization source with SFC separation has been reported. The multimodal ionization source ESCi from Waters has been compared with ESI and APCI in terms of SFC peak broadening,^24^ and it was tested for the detection of hexabromocyclododecane diastereomers.^25^ UniSpray (US) was introduced by Waters as an alternative to ESI to enhance the responses for nonpolar analytes. US is an ESI-based ionization source differing in the ionization mechanism as the voltage is applied to the cylindrical stainless steel rod placed in between the capillary and the MS cone, as opposed to the capillary voltage in ESI.^26^ A higher signal intensity resulting in better sensitivity was confirmed for LC.^27^ For SFC, the increase in sensitivity was more analyte dependent.^26^ Again, no application of the SFC method using a US ionization source has been reported.

Although many different SFC methods have been published and the introduction of UC has significantly expanded the spectrum of compounds that can be analyzed, no universal method has been reported that allows the analysis of nonpolar and polar plant metabolites in a single analytical run. The aim of our work was to optimize a universal UHPSFC-MS method for the analysis of plant extracts. The target compounds were selected as representative compounds that are biologically active, pharmaceutically important, and belong among the most studied compounds, and new analytical methods are still needed for their selective and sensitive analysis. We emphasized the high throughput of the analysis, where only one instrument can be used without the need for manual intervention. Thus, in our pioneering study, we tested different column chemistries allowing the retention of compounds with different physicochemical properties and four different ion sources, i.e., ESI, APCI, ESCi, and US. We present here the proof-of-concept UHPSFC-MS approach, which for the first time enables straightforward analysis of compounds ranging from nonpolar terpenes to polar flavonoids and phenolic acids in a single instrument within two injections.

## Experimental Section

### Chemicals and Reagents

Reference standards of 17 terpenes, 3 terpenoic acids, and 20 phenolic compounds used in this study are summarized in Table S1. Pressurized liquid CO_2_ 4.5 grade (99.9995%) was purchased from Messer (Prague, Czech Republic). LC/MS grade methanol (MeOH), ethanol (EtOH), propan-2-ol (IPA), acetonitrile (ACN), and water were provided by VWR International (Prague, Czech Republic). Ammonia 4 mol/L solution in methanol, ammonium formate (99%), and formic acid (99.9%) for LC/MS were purchased from Sigma-Aldrich (Steinheim, Germany).

### Standard Solutions and Plant Extracts

Standard stock solutions at a concentration of 1 mg/mL of all reference standards were prepared by dissolving each compound in acetonitrile. In the case of low solubility in ACN, MeOH or MeOH/ACN mixture in a ratio of 50/50 (v/v) was used for the dissolution (Table S1). Stock solutions of isorhamnetin, ellagic acid, and tamarixetin were prepared at 0.1 mg/mL due to their low solubility in pure organic solvents. The solutions were stored at −20 °C. Pure EtOH was used for further dilution and the preparation of mixed solutions. The conditions for the preparation of plant extracts are listed in the Supporting Information S1.

### Chromatographic Conditions

All experiments were carried out using the supercritical fluid chromatography system Acquity UPC^2^ (Waters, Milford, MA, USA) equipped with a binary pump, an autosampler, a column thermostat, a back pressure regulator (BPR), and a PDA detector. The system was coupled to a triple quadrupole mass spectrometer Xevo TQ-XS (Waters) via a commercially available SFC-MS dedicated pre-BPR splitter device with an additional isocratic or binary pump for makeup solvent delivery (Waters).

Several stationary phases were tested during optimization including Viridis BEH (hybrid silica), Viridis BEH 2-ethylpyridine (2-EP), Torus 2-picolylamine (2-PIC), Viridis HSS C18 SB (C18), Torus 1-amino anthracene (1-AA), all in 3.0 × 100 mm, 1.7 μm, Torus Diol (diol) (3.0 × 50 mm and 100 mm, 1.7 μm), BEH HILIC (2.1 × 100 mm, 1.7 μm), CORTECS HILIC (silica) (3.0 × 100 mm, 1.6 μm), all from Waters, YMC Carotenoid C30 (4.6 × 100 mm, 5 μm) from YMC (Dinslaken, Germany, and porous graphitic carbon (PGC) columns Hypercarb (3.0 × 50 mm and 100 mm, 3.0 μm, Thermo Fisher Scientific Inc., Waltham, MA, USA) and Supel Carbon LC (3.0 × 150 mm, 2.7 μm, Merck KGaA, Darmstadt, Germany). Analyses were carried out using methanolic organic modifiers with or without an additive that included 10 mmol/L ammonia, 5% water, and 10 mmol/L formic acid. Final chromatographic conditions were as follows: *Method 1:* 150 mm Supel Carbon LC column at 60 °C, BPR pressure at 3300 psi (22.75 MPa), flow rate 1.5 mL/min, MeOH as organic modifier in gradient elution: 0% for 1.5 min, 0–40% in 1.5–4.0 min, 40–41% in 4.0–6.0 min, followed by 1.5 min equilibration at starting conditions. *Method 2:* 50 mm Torus Diol column at 20 °C and 5% water in MeOH as organic modifier with specific gradient conditions listed in Table 1. The partial loop with needle overfill injection mode was used to inject 2 and 10 μL samples for methods 1 and 2, respectively. The autosampler was cooled to 5 °C to reduce the evaporation of volatile compounds.

**Table 1:** Chromatographic Conditions of Method 2^a^

time (min)	CO_2_ (%)	OM (%)	flow rate(mL/min)	BPR (MPa)
0	100	0	1.5	13.0
0.5	100	0	1.5	13.0
0.7	97	3	1.5	13.0
5.0	96	4	1.5	13.0
5.5	80	20	1.5	13.0
7.0	80	20	1.5	13.0
8.5	77	23	1.5	13.0
10.0	77	23	1.5	13.0
12.0	30	70	1.5	13.0
13.0	10	90	0.7	10.3
14.0	10	90	0.7	10.3
14.5	100	0	1.5	10.3
15.0	100	0	1.5	13.0
15.5	100	0	1.5	13.0

a OM: organic modifier.

### MS Conditions

The four ionization sources, ESI, APCI, multimodal ESCi, and US, all from Waters were tested. MassLynx Software 4.1 was used for MS control, data acquisition, and processing. Optimization of the ionization sources conditions was carried out using a design of experiment (DoE) approach, followed by the optimization of the makeup solvent composition and flow rate. The makeup solvents tested included pure MeOH and MeOH with various additives, such as water, ammonia, formic acid, and their combinations in different concentrations. The makeup solvent flow rate was tested in the range of 0.0–0.6 mL/min. Selected reaction monitoring (SRM) transitions were determined for each analyte (Supporting Information S2), and their selectivity was verified. MODDE Software 13.0.1 was used to design the experiments for the ion source optimization and subsequent data evaluation. The parameters tested and their respective ranges for each ionization source are listed in Table 2. The DoE were selected as a compromise between the number of experiments required and the power of the study. Three replicates were always included to test the repeatability of the model. The data evaluation included its logarithmic transformation when necessary and the exclusion of outliers to achieve the highest possible linearity (*R*^2^), model validity, predictability (*Q*^2^), and reproducibility. The limits for the applicable model were set according to the MODDE software: *R*^2^ showing model fit >0.5, *Q*^2^ estimating future prediction precision >0.1 for significant model and >0.5 for a good model, reproducibility >0.5. Model validity tests diverse model problems and should be >0.25. However, model validity can be lost for very good models with *Q*^2^ > 0.9 due to high sensitivity in the test or extremely good replicates (>0.9). Therefore, model validity was always carefully checked manually. The critical factors (marked with “!“ in Table 2) were identified as having a significant effect on the model, i.e., contributing more than 50% to the final response. Therefore, these factors were optimized separately, and a second DoE was run.

**Table 2:** Parameters of the Design of Experiment Approach Used to Optimize the Conditions of Ionization Sources^a^

parameters	APCI - 1	APCI - 2	US - 1	US - 2	ESCi - 1	ESI - 2
corona current (μA)	1–30	1–30			1–30	
desolvation gas flow rate (L/h)	300–1000	300– 1000	300–1000	300–1000	500–1200	500–1200
cone gas flow rate (L/h)	150–900	150–900	150–900	150–900	150–900	150–900
nebulizer pressure (bar)	5–7	5–7	5–7	5–7	5–7	5–7
probe temperature (°C)	100–600	100–600				
cone voltage (V)	5–150! (82%)	10–150	5–150! (55%)	5–150! (60%)	5 - 150/5–150	5–150
impactor voltage (kV)			0.5–4	0.5–4		
desolvation temperature (°C)			150–650	150–650	200–600	200–600
capillary voltage (kV)					0.5–5	0.5–5
design	full factorial	CCF	CCF	CCF	CCF	CCF
power (%)	100	83	78	78	84	83
total runs (runs + replicates)	35 (32 + 3)	47 (44 + 3)	29 (26 + 3)	29 (26 + 3)	83 (80 + 3)	47 (44 + 3)
fitted with	MLR	MLR	MLR	MLR	MLR	PLS

a 1, method 1; 2, method 2; APCI, atmospheric pressure ionization; US, UniSpray; ESI; electrospray; ESCi, multimodal ionization source; MLR, multilinear regression; PLS, partial least square; CCF, central composite face; !, critical parameter with the percentage of contribution to the final MS response.

### Quantitative Parameters

Calibration range, limits of detection and quantification (LOD, LOQ), and repeatability were evaluated and compared. Linearity of the SFC-MS methods was tested in range of 0.1–1000 ng/mL using 13 concentration levels. The linearity was determined using the linearity test in Minitab Software and based on the correlation coefficient and analysis of variance with a significance level of 0.05. In addition, residuals, i.e., % error, were calculated as the percentage difference between the true concentration and the concentration back-calculated from the peak area and calibration equation, with an acceptance level of 10%. The lower limit of quantitation (LLOQ) was established as the lowest concentration level with signal/noise >10 and %-error <20%. Finally, a system suitability test was carried out by determining the relative standard deviations (RSD) of retention times and peak areas within 10 injections at 3 different concentration levels. As the repeatability was consistent over the whole tested calibration range, higher concentration levels, i.e., 50, 100, and 500 ng/mL for method 1 and 100, 500, and 1000 ng/mL for method 2 were selected to cover all target analytes even with higher LLOQs.

## Results and Discussion

The aim of this study was to develop a holistic method for the simultaneous analysis of compounds typically found in plant extracts, namely, flavonoids, terpenoic and phenolic acids, and terpenes. These compounds include molecules with a wide range of physicochemical properties, from small lipophilic terpenes to polar flavonoids, as listed in Table S1.

First, a stationary phase had to be selected that would allow the retention of all of the target compounds. We expected that the retention and elution of hydrophilic flavonoids, and especially their glycosylated forms, i.e., rutin, hirsutrin, and hesperidin, would be the main challenge of the UHPSFC-MS method due to the low polarity of the CO_2_-based mobile phase. On the other hand, less polar terpenes should be easily retained and eluted. A systematic column screening was carried out on a set of selected stationary phases, including hybrid silica, 2-EP, C18, 2-PIC, and diol with preferred H-bonding and π–π interactions. A generic gradient from 0 to 45% of organic modifier, i.e., MeOH, 10 mmol/L ammonia in MeOH, 10 mmol/L formic acid in MeOH, and 2% water in MeOH, was tested on all five columns. As expected, the addition of water to the organic modifier had a beneficial effect on the elution of polar flavonoids. However, it was necessary to increase the percentage of organic modifiers up to 58% to enable the elution of all target analytes. Unfortunately, these stationary phases were not able to retain most of the lipophilic terpenes, especially those without hydroxy groups. That was surprising as more lipophilic compounds are commonly analyzed by SFC. However, cymene, limonene, pinene, caryophyllene, and citronellal eluted in the dead volume even when using pure CO_2_. Volatiles containing hydroxy groups were retained on C18, diol, silica, and 2-EP stationary phases but still coeluted in 1 peak near to the dead volume. Therefore, the retention and separation of terpenes became the main challenge. Accordingly, the 1-AA column with dominant π–π interactions was tested, where the higher retention of nonpolar compounds was expected. This hypothesis was confirmed. However, flavonoids were retained to such an extent that they could not be eluted within a reasonable analysis time under any conditions tested.

Thus, a holistic approach to the analysis of a plant extract consisting of two UHPSFC methods carried out on two columns and the same system without any manual intervention was developed and tested as a proof of concept. Indeed, most commercially available SFC instruments use a column manager that allows two or more columns to be connected simultaneously. Thus, it is still possible to analyze a complex sample in just two injections, taking advantage of the two stationary phases with different selectivity.

### UHPSFC Separation of Terpenes: Method 1

The volatile analytes (Table S1) involved several isobaric groups with the same *m*/*z*: (i) eucalyptol, linalool, citronellal, terpineol, and geraniol, *m*/*z* 155.3; (ii) fenchone and citral, *m*/*z* 153.2; (iii) citronellol and menthol, *m*/*z* 157.3. Therefore, the chromatographic separation of these compounds was crucial to enabling their reliable quantification. Nonpolar stationary phases such as C18, C30, and 1-AA seemed to be the most promising for their retention. Unfortunately, the use of stationary phases with alkyl chains resulted in the elution of linalool, citronellal, and eucalyptol in the dead volume. In contrast, pinene, limonene, and cymene eluted in the dead volume on the 1-AA column. The next logical step was to couple the two stationary phases. However, despite careful optimization, no satisfactory separation was achieved.

Therefore, a less typical stationary phase for UHPSFC was tested, namely, the 50 mm PGC. All 17 volatile analytes were sufficiently retained on this column and eluted within 4 min under generic gradient conditions with pure MeOH as an organic modifier, at 40 °C and 13 MPa. However, two problems occurred: (i) poor peak shape of menthol, citronellol, and terpineol and (ii) insufficient separation of linalool and citronellal with the same *m*/*z*. Therefore, a thorough optimization of the organic modifier composition and gradient program was carried out in the next step.

**Figure 1:**
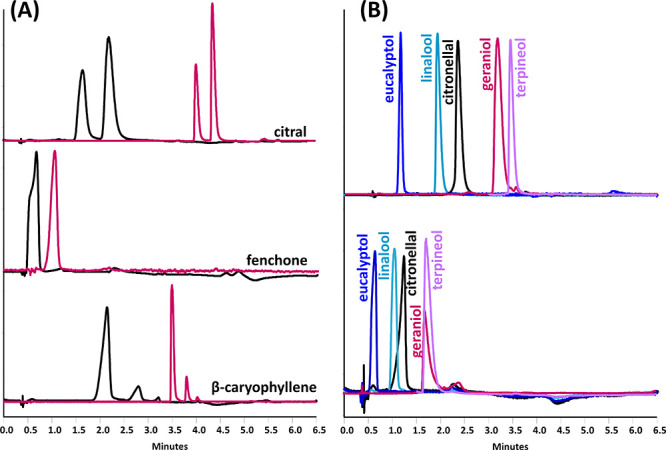
(A) Comparison of peak shapes of volatile terpenes obtained using 3.0 × 100 mm, 5 μm PGC column (in black) and 3.0 × 150 mm, 2.7 μm PGC column (in purple). (B) Comparison of resolution between critical isobaric compounds obtained using a 3.0 × 150 mm, 2.7 μm PGC column (top) and a 3.0 × 100 mm, 5 μm PGC column (bottom).

**Figure 2:**
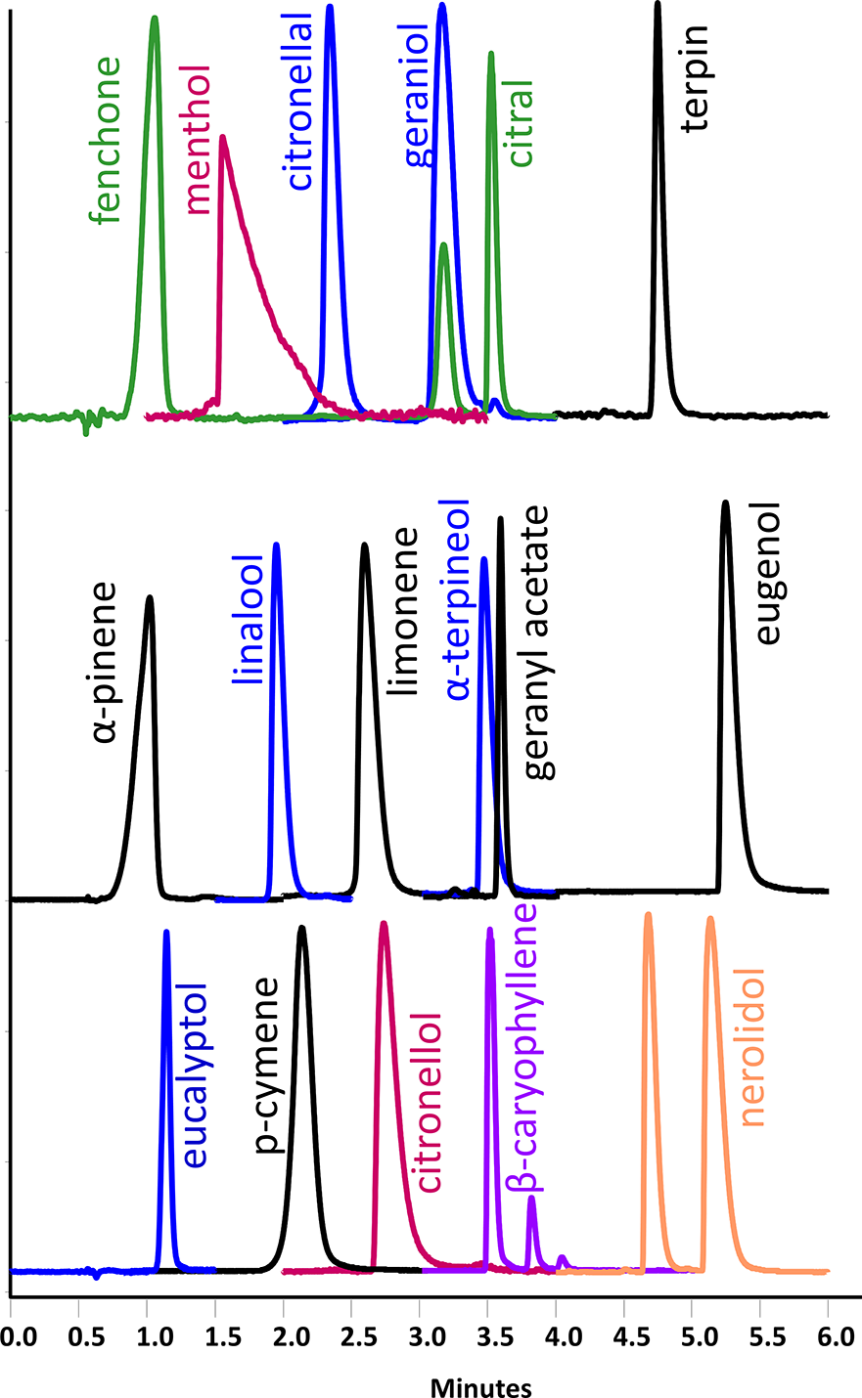
Overlay of UV traces at 211 nm of target terpenes obtained using UHPSFC method 1. For conditions, see Table 1. The isobaric compounds are marked with the same color.

Change of the organic modifier from MeOH to MeOH/ACN, and addition of 2% water did not improve separation, while changing temperature and BPR pressure did positively affect the separation. Indeed, increasing the column temperature improved the resolution between the early eluting peaks, such as eucalyptol, pinene, linalool, cymene, and citronellal. However, the peak shapes of citronellol and terpineol remained unsatisfactory. Increasing the BPR pressure resulted in narrower peaks for all analytes. Therefore, a pressure of 27.5 MPa and a temperature of 60 °C were selected. Even after the careful optimization, the separation of compounds with the same *m*/*z* of 155.3, i.e., linalool, citronellal, eucalyptol, terpineol, and geraniol, was still unsatisfactory. Thus, the originally tested 50 mm PGC column was replaced by a 100 mm column, and the BPR pressure was decreased to 26.2 MPa to avoid exceeding of the system pressure limits. The separation of the critical peaks improved, but baseline separation was not been achieved. The peaks of fenchone, citronellal, menthol, and citral were quite broad (black chromatograms in Figure 1A). Moreover, fenchone, cymene, and eucalyptol eluted in a narrow separation window close to the dead volume making quantification quite difficult (Figure 1A). Therefore, a 150 mm PGC column with smaller particles, 2.7 μm vs 5 μm in the original column, was selected for the final method. The reduction in particle size improved the peak width of some analytes, especially in the case of citral, fenchone, and caryophyllene (Figure 1A). Moreover, eucalyptol and fenchone eluted 30 s after the solvent peak, contrary to the shorter column. The increased column length and smaller particle size allowed baseline separation of linalool, terpineol, and citronellal (Figure 1B). Additionally, a larger surface area (155 m^2^/g) and smaller pore size (200 Å) improved the column capacity and separation efficiency, while higher retention resulted in improved resolution. The BPR pressure had to be reduced to 22.75 MPa to accommodate the higher system pressure caused by the longer column and smaller particles. However, the difference in BPR setting ensured comparable system pressure during analysis using both 100 mm and 150 mm column. The gradient elution time was also increased up to 6 min to allow the elution of nerolidol and eugenol. Other chromatographic conditions remained the same as for the 50 mm PGC column. The final separation of all 17 target volatiles is shown in Figure 2.

In addition to the separation of all target analytes, the developed method was also able to separate isomers of citral and nerolidol (Figure 2). A second peak was also observed for β-caryophyllene. Unfortunately, we were not able to identify the isomers due to the unavailability of pure individual standards.

### UHPSFC Separation of Flavonoids, Phenolic, and Terpenoic Acids: Method 2

Based on the first experiments carried out for all tested compounds, it was obvious that 4 columns, diol, 2-PIC, silica, and 2-EP were able to retain well all target flavonoids and phenolic/terpenoic acids. However, rutin and hirsutrin were strongly retained in all columns, and their elution was impossible within one injection using typical UHPSFC conditions with a maximum of 45% organic modifier. Therefore, the core–shell HILIC column was tested to allow a higher percentage of modifier to be used due to the lower system pressure. Unfortunately, the elution of both rutin and hirsutrin was not possible, even with 50% organic modifier.

The 3.0 mm × 100 mm diol column was selected for further optimization. First, the pressure gradient was tested to allow the use of a higher percentage of the organic modifier, up to 58%. Although the peak shapes were not optimal, it was possible to elute all target analytes, including rutin and chlorogenic acid, using a gradient elution from 0.5 to 58% of MeOH in 15 min and pressure gradient from 13 to 10 MPa between 10.0 and 11.0 min, crossing the boundaries from SFC to UC.

Second, a shorter 50 mm diol column was tested to facilitate a faster elution of polar compounds. Indeed, the shorter column resulted in changes in three main parameters affecting the analysis of polar compounds. (i) The shorter column caused a lower number of interactions between analytes and the stationary phase. Moreover, it also decreased the system pressure. Thus, (ii) wider ranges of BPR pressures and flow rates could be tested to ensure the use of (iii) a higher amount of organic modifier, enabling earlier elution of the analytes. On the shorter column, an analysis with similar selectivity could be carried out within 10 min. However, several problems remained. It was necessary to improve the peak shape of several analytes and to increase selectivity and resolution between isobaric compounds, including compounds with (i) *m*/*z* 455.4–ursulic acid, oleanolic acid, betulinic acid; (ii) *m*/*z* 315.1–isorhamnetin, tamarixetin; (iii) *m*/*z* 285.2–kaempferol, luteolin; (iv) *m*/*z* 289.3–epicatechin, catechin; (v) *m*/*z* 609.2–rutin, hesperidin, and (vi) *m*/*z* 301.0–ellagic acid, quercetin, and hesperetin. Due to the high polarity of the selected compounds, the addition of water to the organic modifier was evaluated. Peak shapes and resolution improved with an increasing percentage of water. However, separation of the gas and liquid phases occurred at 8% water in MeOH. The addition of other additives to the organic modifier including different concentrations of formic acid and ammonia had no beneficial effect on the separation. Therefore, 5% water in methanol was selected as the optimal organic modifier.

**Figure 3:**
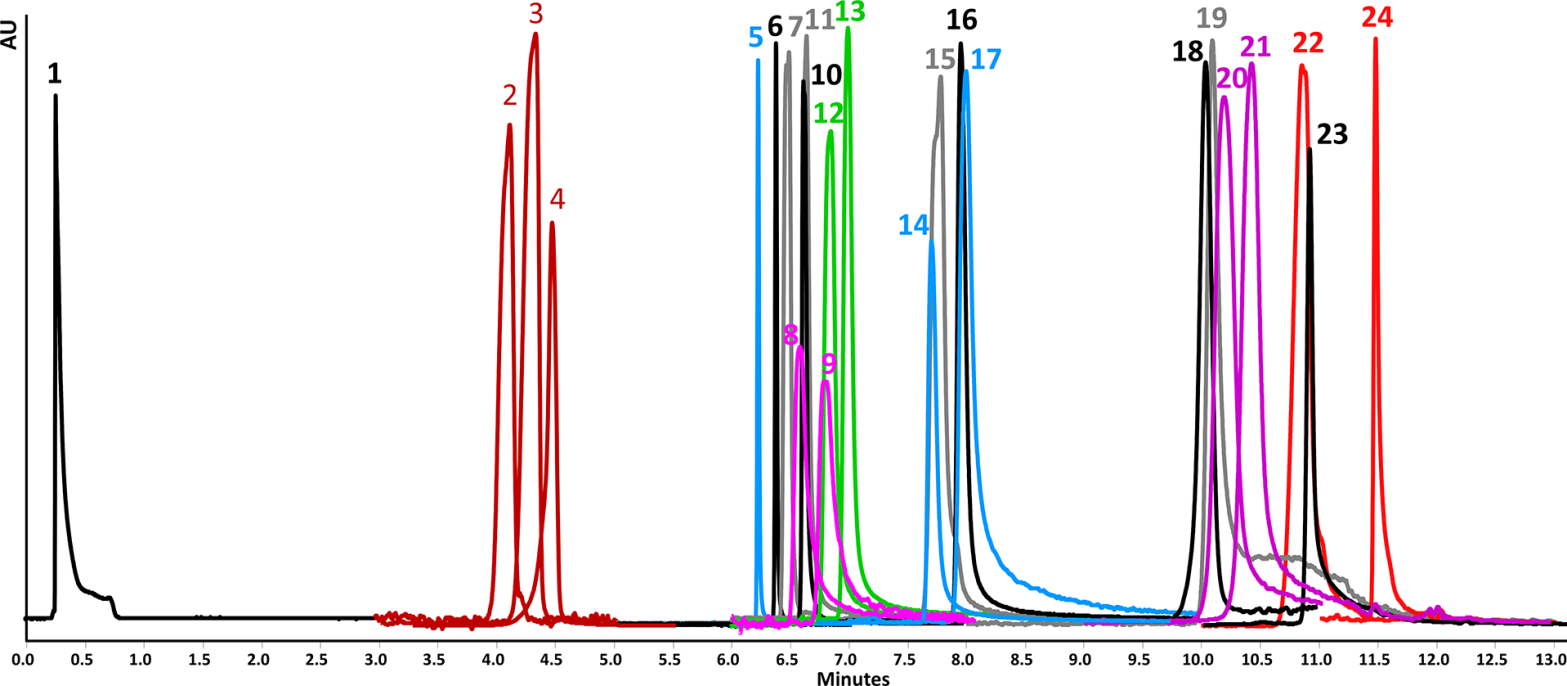
Overlay of UV traces of the target flavonoids and phenolic and terpenoic acids obtained by UHPSFC method 2 with the conditions listed in Table 1. 1—summary peak of terpenes, 2—betulinic acid, 3—oleanolic acid, 4—ursulic acid, 5—hesperetin, 6—naringenin, 7—apigenin, 8—isorhamnetin, 9—tamarixetin, 10—caffeic acid, 11—protocatechuic acid, 12—kaempferol, 13—luteolin, 14—ellagic acid, 15—taxifolin, 16—gallic acid, 17—quercetin, 18—phloridzin, 19—quercitrin, 20—epicatechin, 21—catechin, 22—hesperidin, 23—hirsutrin, 24—rutin. The isobaric compounds are marked with the same color.

The effect of temperature and BPR pressure on the separation was then investigated in the range of 20–60 °C and 10.3–17.2 MPa, respectively. Changing the BPR pressure had no visible effect, but a lower temperature, i.e., 20 °C, resulted in slightly better separation of the critical group of terpenoic acids, i.e., ursulic, oleanolic, and betulinic acid. The fine-tuning included a detailed optimization of the gradient, resulting in a specific gradient program listed in Table 2 with several isocratic steps, gradient of organic modifier and BPR pressure, and different flow rates. The starting point with pure CO_2_ allowed the detection of a composite peak of terpenes (Figure 3). Although it was not possible to separate these compounds, a precursor ion scan using typical fragments of terpenes with *m*/*z* of 135, 137, and 155 could be used for tentative estimation of the terpene content. However, terpenes without this fragmentation pattern could be missed in this case. Thus, this composite peak of terpenes could be advantageous especially in the case of 2D-SFC where the first 2 min of the eluent could be immediately directed to the second column. Increasing the organic modifier to 70% at the end of the gradient program was necessary to elute rutin and hesperidin. This was followed by an increase to 90% mainly due to the high carry-over that occurred when analyzing higher concentrations of target compounds. The additional wash step was also designed to ensure adequate rinsing of the column during the analysis of plant extracts containing even more polar compounds. In addition, a special mixture of MeOH/ACN/IPA/water with 1% formic acid had to be used as a strong solvent for washing the needle to reduce the carry-over.

The overlay of UV traces of each target compound is shown in Figure 3. Using the UC method, it was possible to elute and separate all flavonoids and phenolic and terpenoic acids with sufficient resolution. The only compounds not separated perfectly were 3 epimeric pairs: (i) betulinic and oleanolic acid (resolution (Rs) 0.74), (ii) isorhamnetine and tamarixetine (Rs 0.74), and (iii) and catechin and epicatechin (Rs 0.78). The Rs for the rest of the critical pairs were 1.15 for oleanolic acid/ursulic acid, 1.02 for kaempferol/luteolin, 1.56 for ellagic acid/quercetin, and 5.76 for hesperidin/rutin. Most compounds eluted in narrow symmetrical peaks, except for quercetin and quercitrin. Here, faster changes in gradient composition resulted in better peak shapes but also compromised the resolutions between the remaining analytes. The final conditions were therefore selected as a compromise between the peak shape, resolution, and analysis time. However, even these nonbaseline separations of epimeric pairs and nonperfect peak shapes were sufficient for reliable and reproducible qualitative and quantitative analysis of target compounds even in *Eucalyptus sp.* extracts.

### MS Optimization

In the first step, selected reaction monitoring (SRM) transitions were optimized for all analytes. Subsequently, collision energies were optimized and are listed in Supporting Information S2.

#### Electrospray and Multimodal Ionization

ESI, as the most widely used ionization source in SFC-MS, was selected as the ionization source of the first choice due to its universal and widely available nature. The optimization of ESI-MS parameters was carried out using DoE as listed in Table 1. The power of the model for method 2 was 83% with *R*^2^ 0.80, *Q*^2^ 0.65, validity 0.32, and repeatability 0.99. The proposed final conditions are given in Table 3.

**Table 3:** Optimal MS Conditions for All Tested Ionization Sources^a^

final conditions	APCI - 1	APCI - 2	US - 1	US - 2	ESCi - 1	ESI - 2
corona current (μA)	25	30			2	
desolvation gas flow rate (L/h)	1000	970	1000	700	720	500
cone gas flow rate (L/h)	350	150	900	300	280	250
nebulizer pressure (bar)	5	7	5	5	5	6
probe temperature (°C)	450	560				
cone voltage (V)	10	145			150/5	5
impactor voltage (kV)			4	2.5		
desolvation temperature (°C)			500	650	350	200
capillary voltage (kV)					1	0.5

a 1—method 1; 2—method 2.

Unfortunately, ESI was not suitable for the ionization of terpenes, especially when the molecules did not contain any hydroxy groups. The peaks of cymene, limonene, and pinene were observed with high sensitivity in UV detection, but no molecular ions were detected in ESI-MS. Therefore, a multimodal ionization source combining ESI and APCI (ESCi) was tested in the next step. The addition of a corona charge enabled or improved the ionization of compounds without functional groups. Thus, ESCi was optimized instead of ESI for method 1, and the goal of a holistic 2-injection method remained unchanged as the software allows switching between ESI and ESCi ionization without the need to manually change the instrumentation. DoE approach with the parameters listed in Table 1 was carried out to optimize the ESCi source parameters. The power of the model was 84%, with *R*^2^ 0.96, *Q*^2^ 0.94, validity 0.57, and repeatability 0.99. The final setting is shown in Table 3 and was used for the subsequent optimization of the makeup solvent composition. Here, it should be emphasized that the same ion source geometry cannot be available for all instrumentations on the market. Thus, careful tuning of the parameters depending on the ionization source geometry has to be carried out for individual applications.

The tested makeup solvents included 10 mmol/L formic acid in MeOH, 10 mmol/L ammonia in MeOH, 1% water in MeOH, and MeOH containing the combination of all 3 additives (abbreviated as MU3). Since only one makeup solvent had to be selected for both SFC-MS methods to avoid manual intervention during the 2-injection analysis, a compromise had to be found. The flow rate of the makeup solvent was critical especially for volatiles in method 1. Indeed, flow rates up to 0.3 mL/min increased the sensitivity for terpenes with a hydroxy group. In contrast, no addition of makeup solvent, i.e., 0 mL/min, was beneficial for terpenes without any functional groups as shown in Supporting Information S3. However, the addition of makeup solvent was necessary to mitigate possible precipitation of analytes and to ensure stable flow to the MS. Finally, MU3 was selected at 0.1 and 0.3 mL/min for methods 1 and 2, respectively, which allowed the ionization and detection of both groups of analytes with sufficient sensitivity.

#### Atmospheric Pressure Chemical Ionization

Since the ESCi application resulted in an improvement in the ionization of nonpolar compounds, we expected that the APCI would be even more advantageous not only in ionization but also due to the lower susceptibility to matrix effects, which is beneficial when analyzing complex matrices such as plant extracts.

The parameters tested using the DoE are listed in Table 2. The cone voltage was identified as a critical factor affecting the final response by >82% in the case of method 1. The lower the cone voltage, the higher MS response was observed for all target analytes. Thus, 10 V was selected as the optimal cone voltage and the DoE was repeated. The contributions of the tested factors to the observed responses are listed in Supporting Information S4. The fit of the model used was confirmed by power of the study 100%, *R*^2^ 0.97, *Q*^2^ 0.94, validity 0.57, and reproducibility 0.99. The final conditions are summarized in Table 3. On the other hand, the cone voltage was not a determining factor for method 2 as it only affected the response by 17.6% (Supporting Information S4). The model showed a satisfactory fit with power of the study 83%, *R*^2^ 0.92, *Q*^2^ 0.85, validity 0.40, reproducibility 0.89. Thus, the optimal conditions suggested by the DoE were confirmed in the following experiments and are listed in Table 3.

Higher concentrations of additives in the eluent entering the MS are beneficial for APCI-MS due to the different ionization mechanism.^14^ Therefore, several makeup solvent compositions and flow rates were tested including 10, 20, and 50 mmol/L ammonia in MeOH and MU3 at 0.1, 0.2, 0.3, and 0.4 mL/min. The UHPSFC-APCI-MS setup used the same interface with a splitter and a sheath pump as in the case of ESI/ESCi. However, it is not preferable for APCI ionization, as it is a mass-dependent ionization source. Thus, the lowest flow rate of makeup solvent, i.e., the lowest dilution ratio, resulted in the highest MS responses, similar to ESCi, as shown in Supporting Information S3. Finally, the use of MU3 at 0.1 mL/min was selected as optimal for both methods. Overall, the APCI source was particularly suitable for lipophilic volatile terpenes and terpenoic acids. However, the sensitivity for polar flavonoids, especially routine and hesperidin, was insufficient in the 700–1000 ng/mL range even after careful optimization of all parameters.

#### UniSpray Ionization

The optimization was again carried out using the DoE approach, with the tested parameters listed in Table 2. The power of the model was 78% for both methods with *R*^2^ 0.82 and 0.94, *Q*^2^ 0.58 and 0.85, validity 0.46 and 0.77, and reproducibility 0.96 and 0.93 for methods 1 and 2, respectively. The selected optimum conditions are listed in Table 3. The same makeup solvent compositions as for other ionization sources were tested. An ammonia-based makeup solvent resulted in a good sensitivity for most of the target compounds. The addition of water to the makeup solvent had a positive effect on the responses of nerolidol, menthol, and most of the flavonoids and formic acid decreased the MS responses by 20–60% for most compounds. The MU3 was again selected as a compromise, enabling the detection of all compounds with satisfactory sensitivity (Supporting Information S3). The flow rate of the makeup solvent had almost no effect on the MS responses (Supporting Information S3) in method 2. Thus, 0.3 mL/min was selected for the final method. However, lower flow rates of makeup solvent increased the responses of most of the terpenes and 0.1 mL/min was selected for method 1.

In general, US was beneficial for the sensitivity of all compounds, except for cymene, limonene, and pinene. Since these compounds do not contain any functional groups, their ionization in the US was not possible even after testing of all optimizable parameters within instrumentation limits.

### Comparison of Ionization Sources and Quantitative Aspects

Finally, repeatability of retention time (*t*_R_) and peak areas, calibration range, lower and upper LOQ (LLOQ, ULOQ), and specificity of methods 1 and 2 were tested using the optimize conditions. While method 1 targeting volatile terpenes showed very good sensitivity with LLOQ in the range of 0.01–1 ng/mL for APCI, it was clear that APCI is not at all suitable for the detection of polar flavonoids and phenolic acids, as their LLOQs were usually >500 ng/mL (Supporting Information S5). US enabled reliable detection of all flavonoids and phenolic acids with low LLOQ (0.5–20 ng/mL) and symmetrical narrow peaks. However, it did not allow the detection of pinene, limonene, and cymene with the LLOQ for terpenoic acids about three times lower with APCI than with US (2 vs 7 ng/mL). Slightly lower sensitivity was obtained with ESI than with US, but the LLOQs for most of the compounds were comparable between these two ionization sources.

The repeatability of *t*_R_ and peak areas determined as RSD were within acceptable limits, i.e., < 1.5% for *t*_R_ and <10% for peak areas at 3 concentration levels tested with ESI/ESCi. The same was true for US and 1 concentration level measured by APCI.

However, the main advantage of the ESI source was the possibility to easily change it to the multimodal ionization source ESCi, which enabled detection of all targeted terpenes. Indeed, the LLOQ achieved with APCI was 10–20 times lower than with ESCi for volatile compounds, while US showed slightly better sensitivity than ESI for polar compounds. However, the combined ESI/ESCi sources represented a compromise that enabled simultaneous analyses of terpenes and flavonoids without the need for manual change in instrumentation.

### Application of UHPSFC-ESI/ESCi-MS/MS Method on *Eucalyptus* Extracts

The final method was used for the analysis of *Eucalyptus sp*. extracts prepared by supercritical fluid extraction (SFE). EtOH was used as an organic modifier during the SFE extraction. Thus, EtOH was selected also as an injection solvent for the analytical method despite its slightly negative effect on peak shape compared to acetonitrile.

**Figure 4:**
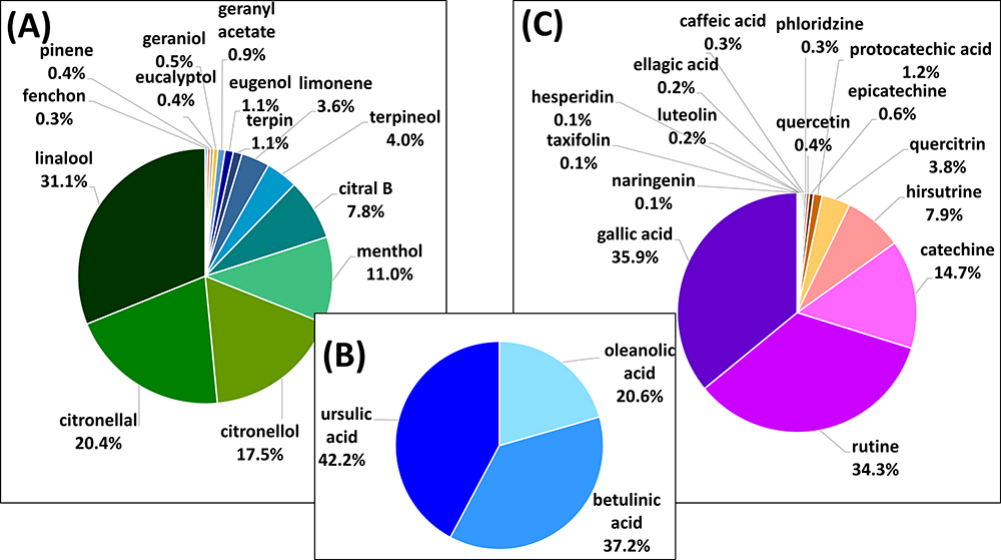
Comparison of the percentage abundance of targeted analytes, i.e., (A) terpenes, (B) terpenoic acids, (C) flavonoids and phenolic acids, detected in SFE extract of *Eucalyptus sp.* detected using UHPSFC-ESI/ESCi-MS/MS approach.

Based on the conditions (Supporting Information S1), different concentrations of flavonoids and volatiles were expected in these extracts. As no internal standards were used for the analysis of plant extracts, a full validation including determination of matrix effects needs to be carried out in future studies. Thus, only semiquantitation of target analytes was possible. Both pure and diluted extracts were measured to allow a semiquantification of the target analytes as concentration of many of the target compounds exceeded the ULOQ of the UHPSFC-ESI/ESCi-MS/MS method. Overall, > 90% of the compounds detected were terpenoic acids, followed by 9% of flavonoids/phenolic acids and only 0.4% of terpenes. Linalool, citronellal, citronellol, and menthol were among the most abundant terpenes (Figure 4A). The three target terpenoic acids were detected in similar concentrations in the extracts (Figure 4B). Gallic acid, rutin, catechin, and hirsutrin were among the most abundant flavonoids and phenolic acids (Figure 4C). The observed amount of target analytes is strongly affected by the conditions used in SFE. However, this optimization was not carried out within the scope of this study. Thus, a comparison of three extracts obtained using three different SFE conditions is shown in Supporting Information S6 to demonstrate the dependence between the observed concentration of target analytes and SFE conditions.

## Conclusions

UHPSFC-MS/MS is an interesting alternative to LC and GC methods, permitting the analysis of a wide range of compounds with a single instrument. Unfortunately, a single holistic method for the simultaneous analysis of terpenes and polar flavonoids remains unattainable due to two main challenges. No tested stationary phase enabled the retention and elution of both groups of analytes. However, new stationary phases are being introduced to the market every year, which is promising. Second, a universal ionization source combining GC and LC approaches is required for the simultaneous analysis of small lipophilic terpenes and polar glycosylated flavonoids.

Despite these challenges, we were able to develop a two-injection approach for complex plant analysis as a proof-of-concept based on the ability of the UHPSFC to cover supercritical, subcritical, enhanced-liquid, and liquid conditions within one instrument. Here, volatile terpenes are measured using a PGC stationary phase within supercritical to subcritical conditions achieved by adding up to 40% of MeOH to CO_2_. More polar phenolic and terpenoic acids and flavonoids are then analyzed on short diol column with gradient from 0 to 90% MeOH with addition of 5% water.

The APCI source proved to be beneficial in the case of lipophilic volatiles when compared to ESI, but it was not possible to detect flavonoids using APCI. Conversely, US resulted in higher sensitivity for flavonoids, but similar to ESI, it was not possible to detect terpenes without hydroxy groups. The finally selected multimodal ESCi source was able to detect all targeted volatiles although the sensitivity was significantly lower than in the case of APCI. However, it was possible to switch from ESCi to ESI without any need for manual intervention, which was crucial for the applicability of the developed approach.

Finally, the quantitative aspects of the two developed UHPSFC-ESI/ESCi-MS/MS methods were determined, and the approach was successfully applied for the analysis of SFE extract from *Eucalyptus sp*. The novel approach can be beneficial for analysis of complex samples containing analytes with a wide range of physicochemical properties.

## Supporting Information Available

Experimental conditions of SFE extraction and list of analytes; optimized SRM transitions for all target analytes; graphs summarizing makeup solvent optimization; design of experiments—contribution of tested parameters to the observed MS response; comparison of lower limits of quantification; and comparison of analytes concentration in different SFE extracts (PDF)
