# Supplementary material for: Ultrahigh-Performance Supercritical Fluid Chromatography–Multimodal Ionization–Tandem Mass Spectrometry as a Universal Tool for the Analysis of Small Molecules in Complex Plant Extracts
Source: Anal Chem. 2024 Feb 1;96(7):2840–8. doi: 10.1021/acs.analchem.3c03599 (PMC10882571; doi:10.1021/acs.analchem.3c03599)
Supplement: Supplementary file 1 — ac3c03599_si_001.pdf [file ac3c03599_si_001.pdf]

Supporting Information: Ultra-High Performance Supercritical Fluid Chromatography-Multimodal Ionization-Tandem Mass Spectrometry as a Universal Tool for Analysis of Small Molecules in Complex Plant Extracts

Kateřina Plachká, Veronika Pilařová, Štefan Kosturko, Frantisek Svec, Lucie Nováková*

Department of Analytical Chemistry, Faculty of Pharmacy in Hradec Králové, Charles University, Heyrovského 1203, 500 05 Hradec Králové, Czech Republic

* Email: nol@email.cz (Lucie Nováková)

Table of Content:

S1. Experimental part

S1a: Supercritical Fluid Extraction

Table S1: List of analytes, including vendor, purity, and physicochemical properties.

S2. SRM transitions

Table S2: SRM transitions selected for each target analyte together with their optimal collision energy.

S3. Make-up solvent optimization

Figure S3a: Comparison of MS responses obtained for selected analytes using ESCi/ESI and various make-up solvents.

Figure S3b: Comparison of MS responses obtained using method 1 with ESCi source and various flow rates of make-up solvent, 0 (blue), 0.05 (purple), 0.1 (pink), 0.2 (orange), 0.3 (green), 0.4 (yellow), and 0.5 (red) mL/min.

Figure S3c: Comparison of MS responses obtained using APCI source and various make-up solvents.

Figure S3d: Comparison of MS responses obtained using method 1 with APCI source and various flow rates of make-up solvent, i.e., 0.1 (blue), 0.2 (red) , 0.3 (green), and 0.4 (yellow) mL/min.

Figure S3e: Comparison of MS responses obtained using method 2 with UniSpray source using various make-up solvents.

Figure S3f: Comparison of MS responses obtained using method 2 with UniSpray source and various flow rates of make-up solvent, i.e., 0.1 (blue), 0.2 (red) , 0.3 (green), 0.4 (yellow), and 0.5 (purple) mL/min.

S4. Design-of-experiments

Table S4: Contribution of tested parameters to the observed MS response using different ionization sources.

S5. Lower limits of quantification

Table S5: Comparison of lower limits of quantification in ng/mL achieved using different ionization sources.

S6. Comparison of different SFE extracts

Figure S6: Comparison of concentrations of target analytes observed in three *Eucalyptus sp.* extracts obtained using three different SFE conditions. Extract 1: CO_2/EtOH 50/50, temperature 50 °C, pressure 16 MPa. Extract 2: neat CO_2, temperature 50 °C, pressure 30 MPa. Extract 3: neat CO_2, temperature 50 °C, pressure 16 MPa.

S1. Experimental part

S1a. Supercritical Fluid Extraction (SFE)

SFE was carried out using SFE system (Waters MV-10, Milford, MA, USA) comprising a fluid delivery module for pumping CO_2 and the co-solvent, an oven holding the extraction vessels, an automated back pressure regulator (BPR), and a fraction collector module. The heads of the CO_2 pump were cooled using a chiller operated at 5 °C. After each extraction the SFE system was flushed with CO_2/co-solvent for 5 min followed by neat CO_2 to remove residual co-solvent from the system. The system was controlled by ChromScopeTM soft-ware (Waters, Milford, MA, USA). 1 g of dried, milled, and homogenized sample was placed in 5 mL stainless steel extraction vessel between two layers of 3 mm glass beads.

The extraction was carried out using following conditions: CO_2/EtOH 50/50, temperature 50 °C, pressure 16 MPa.

Dynamic extraction mode was used in all cases with a flow rate of 2 mL/min controlled as a volumetric ratio between CO_2 and ethanol as a co-solvent. Extraction time was set on 10 min. 1 mL aliquot was directly placed to total recovery microvials and analyzed by optimized SFC-MS methods.

Table S1: List of analytes, including vendor, purity, and physicochemical properties. Summary formula, LogP, pKa, and H acceptors/donors were obtained on SciFinder database. Structure and exact mass were acquired using ChemDraw software.

ANALYTE	VENDOR	PURITY [%]	FORMULA	EXACT MASS [DA]	Log P	pKa	H ACCEPTORS/ H DONORS	STRUCTURE
**1,8-CINEOLE (EUCALYPTOL)**	Sigma-Aldrich	99.3	$C_{10}H_{18}O$	154.1358	2.795±0.267	N/A	1/0	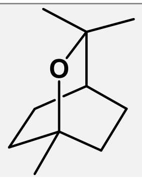
**CITRAL**	Sigma-Aldrich	≥ 97	$C_{10}H_{16}O$	152.1201	3.127±0.359	N/A	1/0	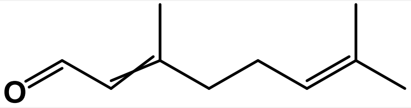
**CITRONELLAL**	Sigma-Aldrich	95.9	$C_{10}H_{18}O$	154.1358	3.297±0.259	N/A	1/0	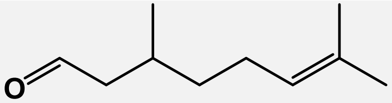
**CITRONELLOL**	Sigma-Aldrich	99.5	$C_{10}H_{20}O$	156.1514	3.239±0.235	15.13±0.10	1/1	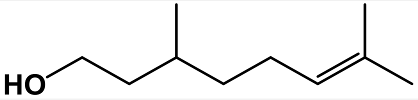
**EUGENOL**	Sigma-Aldrich	99.7	$C_{10}H_{12}O_2$	164.0837	2.403±0.236	10.29±0.18	2/1	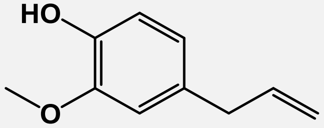
**FENCHONE**	Sigma-Aldrich	≥ 99.5	$C_{10}H_{16}O$	152.1201	2.089±0.300	N/A	1/0	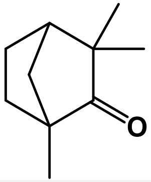
**GERANIOL**	Sigma-Aldrich	98.7	$C_{10}H_{18}O$	154.1358	2.942±0.271	14.45±0.10	1/1	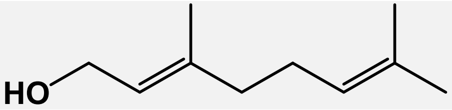
**GERANYL ACETATE**	Sigma-Aldrich	98.3	$C_{12}H_{20}O_2$	196.1463	3.904±0.315	N/A	2/0	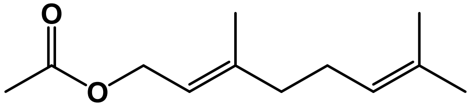

**LIMONENE**	Sigma-Aldrich	99.5	$C_{10}H_{16}$	136.1252	4.552±0.241	N/A	0/0	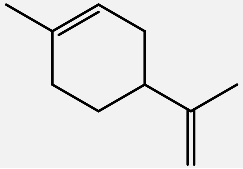
**LINALOOL**	Sigma-Aldrich	98.9	$C_{10}H_{18}O$	154.1358	2.795±0.263	14.51±0.29	1/1	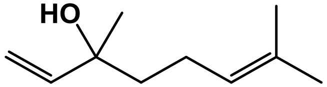
**MENTHOL**	Sigma-Aldrich	99.3	$C_{10}H_{20}O$	156.1514	3.216±0.204	15.30±0.60	1/1	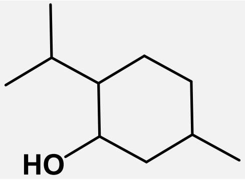
**NEROLIDOL**	Sigma-Aldrich	99.3	$C_{15}H_{26}O$	222.1984	4.682±0.295	14.44±0.29	1/1	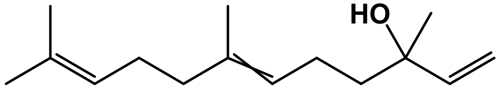
**p-CYMENE**	Sigma-Aldrich	99.7	$C_{10}H_{14}$	134.1096	4.014±0.189	N/A	0/0	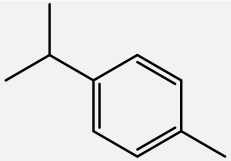
**T-TERPIN**	Sigma-Aldrich	99.5	$C_{7}H_{14}O$	114.1045	1.056±0.242	15.18±0.29	2/2	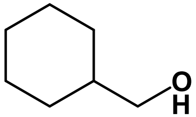
**α-PINENE**	Sigma-Aldrich	99.5	$C_{10}H_{16}$	136.1252	4.321±0.237	N/A	0/0	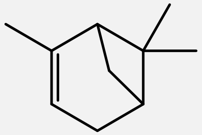
**α-TERPINEOL**	Sigma-Aldrich	98	$C_{10}H_{18}O$	154.1358	2.708±0.236	15.09±0.29	1/1	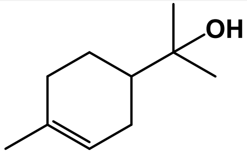

**β-CARYOPHYLLENE**	Sigma-Aldrich	N/A	$C_{15}H_{24}$	204.1878	6.416±0.248	N/A	0/0	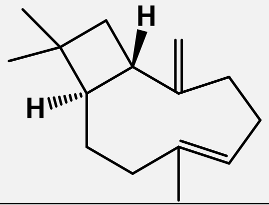
**BETULINIC ACID**	Sigma-Aldrich	≥99.3	$C_{30}H_{48}O_3$	456.3603	7.38	4.75	3/2	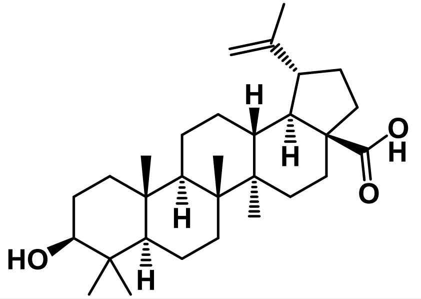
**OLEANOLIC ACID**	Sigma-Aldrich	≥99.9	$C_{30}H_{48}O_3$	456.3603	7.47	N/A	3/2	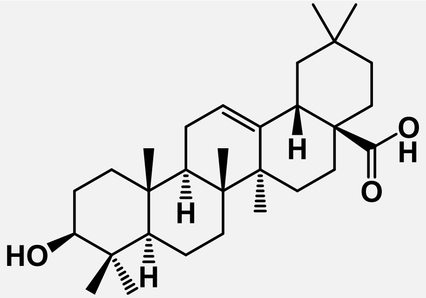
**URSOLIC ACID**	MedChem Express	≥98	$C_{30}H_{48}O_3$	456.3603	7.33	4.74	3/2	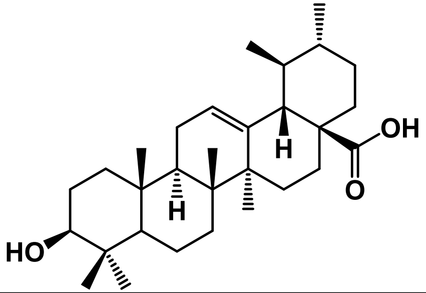

**APIGENIN**	Sigma-Aldrich	99	$C_{15}H_{10}O_{5}$	270.0528	2.127±0.452	6.53±0.40	5/3	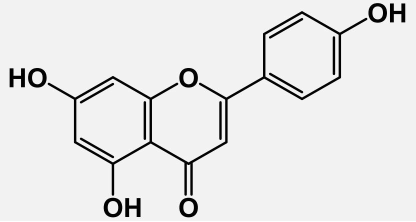
**CAFFEIC ACID**	Sigma-Aldrich	99.4	$C_{9}H_{8}O_{4}$	180.0423	0.663±0.286	4.58±0.10	4/3	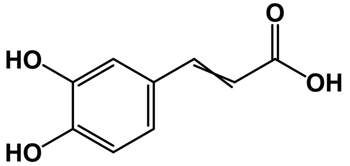
**CATECHIN**	Sigma-Aldrich	99	$C_{15}H_{14}O_{6}$	290.0790	0.610±0.454	9.54±0.10	6/5	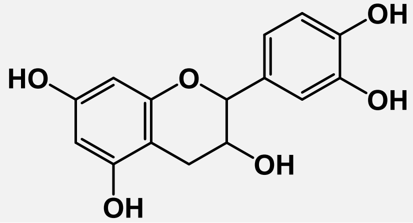
**ELLAGIC ACID**	Sigma-Aldrich	97.4	$C_{14}H_{6}O_{8}$	302.0063	0.239±1.553	5.02±0.20	8/4	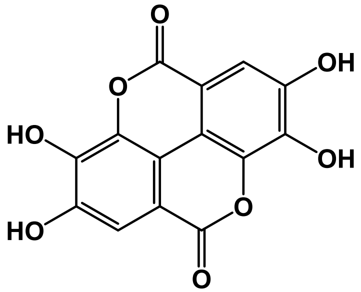
**EPICATECHIN**	Sigma-Aldrich	96.1	$C_{15}H_{14}O_{6}$	290.0790	0.610±0.454	9.54±0.10	6/5	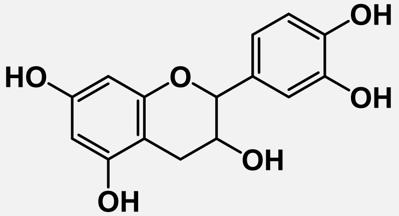

**GALLIC ACID**	Sigma-Aldrich	100	$C_7H_6O_5$	170.0215	0.531±0.325	4.33±0.10	5/4	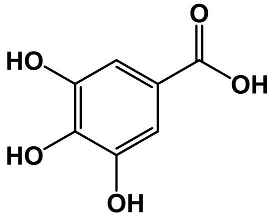
**HESPERETIN**	Sigma-Aldrich	99	$C_{16}H_6O_6$	304.0947	1.938±0.471	7.49±0.40	6/3	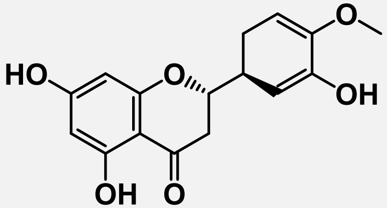
**HESPERIDIN**	Sigma-Aldrich	95.9	$C_{28}H_{34}O_{15}$	610.1898	-1.212±0.819	7.15±0.40	15/8	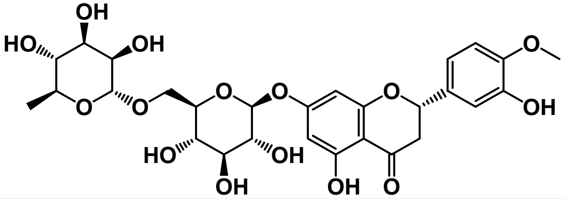
**HIRSUTRIN**	Sigma-Aldrich	N/A	$C_{21}H_{20}O_{16}$	464.0955	-0.111±1.370	6.17±0.40	12/8	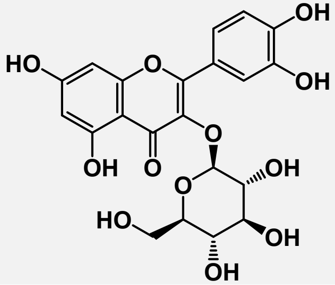
**ISORHAMNETIN**	Extrasynthese	≥99	$C_{16}H_{12}O_7$	316.0583	2.787±0.822	6.31±0.40	7/4	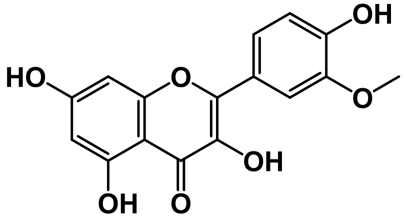

**KAEMPFEROL**	Sigma-Aldrich	97.8	$C_{15}H_{10}O_6$	286.0477	2.685±0.812	6.34±0.40	6/4	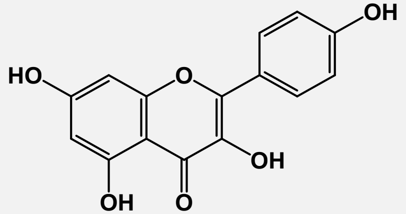
**LUTEOLIN**	Sigma-Aldrich	99.4	$C_{15}H_{10}O_6$	286.0477	2.695±0.554	6.50±0.40	6/4	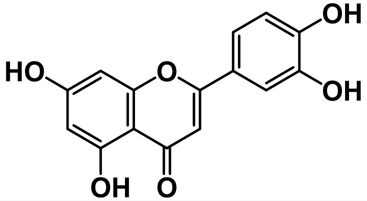
**NARINGENIN**	Sigma-Aldrich	98.9	$C_{15}H_{12}O_5$	272.0685	2.628±0.406	7.52±0.40	5/3	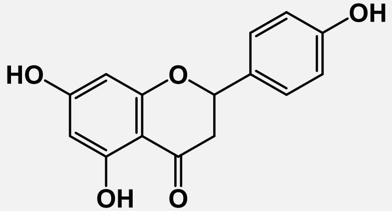
**PHLORIDZIN**	Sigma-Aldrich	99	$C_{21}H_{24}O_{10}$	436.1369	-0.365±0.385	7.15±0.40	10/7	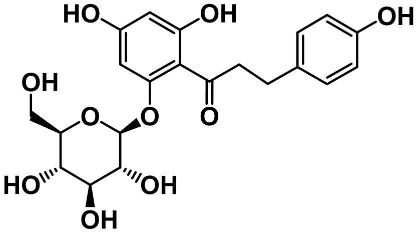
**PROTOCATEC HUIC ACID**	Sigma-Aldrich	99.9	$C_7H_6O_4$	154.0266	1.010±0.237	4.45±0.10	4/3	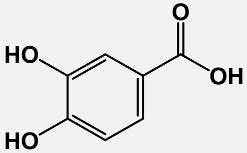

**QUERCETIN**	MedChem Express	≥98	$C_{15}H_{10}O_7$	302.0427	1.989±1.075	6.31±0.40	7/5	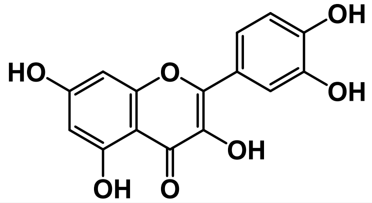
**QUERCITRIN**	MedChem Express	99.24	$C_{21}H_{20}O_{12}$	448.1006	0.579±1.358	6.17±0.40	11/7	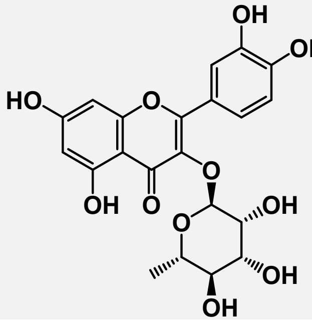
**RUTIN**	Extrasynthese	≥99	$C_{27}H_{30}O_{16}$	610.1534	-0.903±1.416	6.17±0.40	16/10	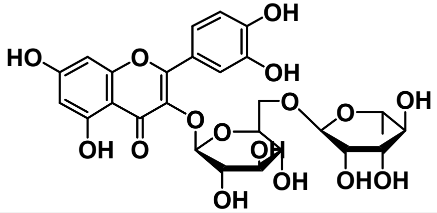
**TAMARIXETIN**	Extrasynthese	≥98	$C_{16}H_{12}O_7$	316.0583	2.670±0.822	6.31±0.40	7/4	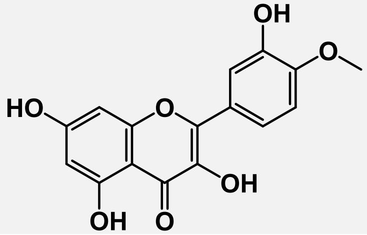
**TAXIFOLIN**	MedChem Express	≥99.5	$C_{15}H_{12}O_7$	304.0583	1.569±0.555	7.39±0.60	7/5	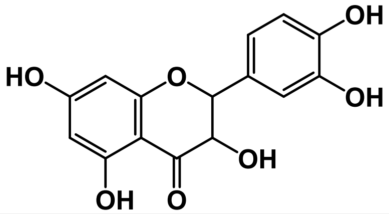

S2. SRM transitions

Table S2: SRM transitions selected for each target analyte together with their optimal collision energy. RT – retention time, CE – collision energy, ESI – electrospray ionization, ESCi – multimodal ionization

ANALYTE	RT (MIN)	IONIZATION MODE	SRM	CE (eV)
**1,8-CINEOLE (EUCALYPTOL)**	1.101	ESCi$^+$	155.3 > 137.2	10
**CITRAL**	3.175 + 3.525	ESCi$^+$	153.1 > 71.1	10
**CITRONELLAL**	2.347	ESCi$^+$	155.3 > 111.2<br>155.3 > 94.97	10
**CITRONELLOL**	2.737	ESCi$^+$	157.3 > 83.1<br>157.3 > 68.8	20
**EUGENOL**	5.242	ESCi$^+$	164.2 > 149.1<br>164.2 > 132.1	10
**FENCHONE**	1.056	ESCi$^+$	152.9 > 135.2<br>152. 9 > 109.1	10
**GERANIOL**	3.168	ESCi$^+$	155.3 > 137.3	10
**GERANYL ACETATE**	3.539	ESCi$^+$	196.2 > 181.2<br>181.2 > 163.2	10
**LIMONENE**	2.590	ESCi$^+$	136.9	x
**LINALOOL**	1.945	ESCi$^+$	137.0 > 81.1	10
**MENTHOL**	1.668	ESCi$^+$	171.0 > 135.0<br>157.3	10
**NEROLIDOL**	4.682 + 4.970	ESCi$^+$	205.2 > 121.1<br>205.2 > 135.0	10
**p-CYMENE**	2.102	ESCi$^+$	134.2 > 119.1	10
**T-TERPIN**	4.756	ESCi$^+$	136.9 > 81.0	10
**α-PINENE**	1.02	ESCi$^+$	137.3	x
**α-TERPINEOL**	3.365	ESCi$^+$	155.3 > 137.3<br>137.3 > 71.1	20
**β-CARYOPHYLLENE**	3.499	ESCi$^+$	205.4 > 149.1<br>205.4 > 121.1	10
**BETULINIC ACID**	4.151	ESI$^-$	455.4 > 455.4<br>455.4 > 407.1	10
**OLEANOLIC ACID**	4.32	ESI$^-$	455.4 > 455 4<br>455.4 > 407.1	10
**URSOLIC ACID**	4.589	ESI$^-$	455.4 > 455.4<br>455.4 > 407.1	10
**APIGENIN**	6.450	ESI$^-$	268.9 > 224.9<br>268.9 > 200.9	20
**CAFFEIC ACID**	6.621	ESI$^-$	178.9 > 135.0	20
**CATECHIN**	10.398	ESI$^-$	289.3 > 203.2<br>289.3 > 178.9	20
**ELLAGIC ACID**	7.771	ESI$^-$	301.0 > 163.1	10
**EPICATECHIN**	10.171	ESI$^-$	289.3 > 203.2<br>289.3 > 178.9	5
**GALLIC ACID**	7.965	ESI$^-$	168.9 > 124.9	10
**HESPERETIN**	6.201	ESI$^-$	301.0 > 241.9<br>301.0 > 199.1	20
**HESPERIDIN**	10.771	ESI$^-$	609.2 > 301.0	20
**HIRSUTRIN**	10.822	ESI$^-$	462.9 > 300.0	30
**ISORHAMNETIN**	6.576	ESI$^-$	315.1 > 299.9<br>315.1 > 271.0	20
**KAEMPFEROL**	6.851	ESI$^-$	285.2 > 150.9<br>285.2 > 228.9	30
**LUTEOLIN**	7.003	ESI$^-$	285.2 > 175.0<br>285.2 > 199.0	30
**NARINGENIN**	6.391	ESI$^-$	270.9 > 150.8<br>270.9 > 177.1	20
**PHLORIDZIN**	10.047	ESI$^-$	434.9 > 272.9	20
**PROTOCATECHUIC ACID**	6.634	ESI$^-$	153.0 > 108.8	10
**QUERCETIN**	8.071	ESI$^-$	301.0 > 179.0<br>301.0 > 245.0	20

**QUERCITRIN**	10.093	ESI$^-$	446.9 > 300.9<br>446.9 > 348.8	20
**RUTIN**	11.474	ESI$^-$	609.2 > 301.0<br>301.0 > 179.2	30
**TAMARIXETIN**	6.729	ESI$^-$	315.1 > 299.9<br>315.1 > 271.0	20
**TAXIFOLIN**	7.794	ESI$^-$	302.9 > 217.0<br>302.9 > 176.9	20

S3. Make-up solvent optimization

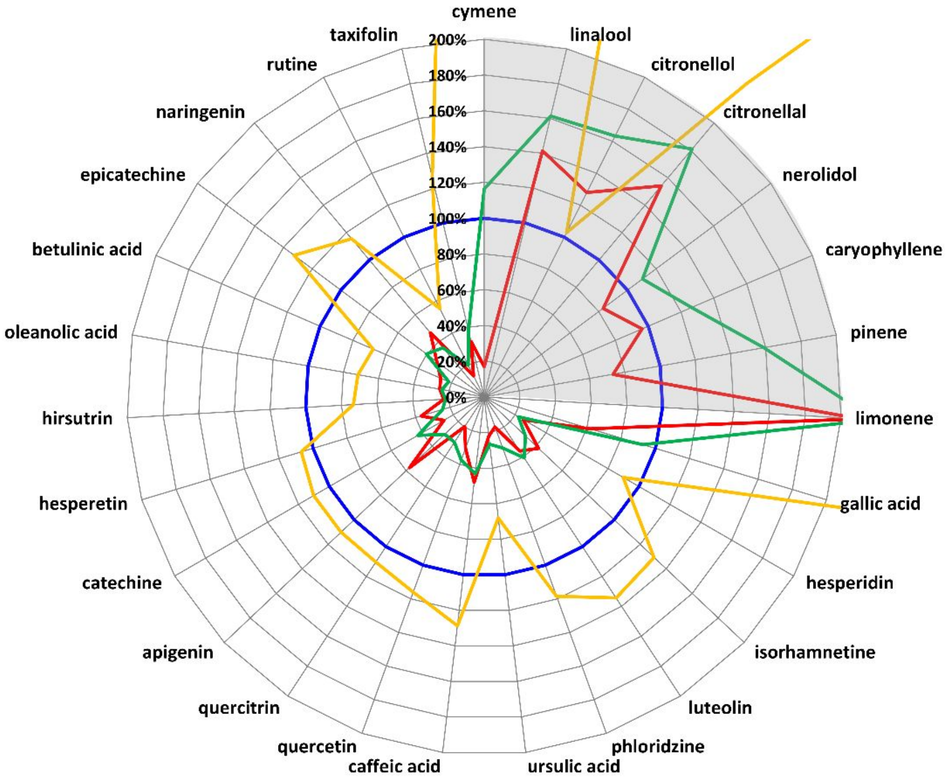

Figure S3a: Comparison of MS responses obtained for selected analytes using ESCi/ESI and various make-up solvents: 10 mmol/L ammonia in methanol (blue), 10 mmol/L formic acid in methanol (red), 10 mmol/L formic acid + 1% water in methanol (green), 10 mmol/L ammonia + 10 mmol/L formic acid + 1% water in methanol (yellow). In gray – volatiles analyzed by method 1 with ESCi source, in white – flavonoids analyzed by method 2 with ESI source. FA – formic acid, MeOH – methanol

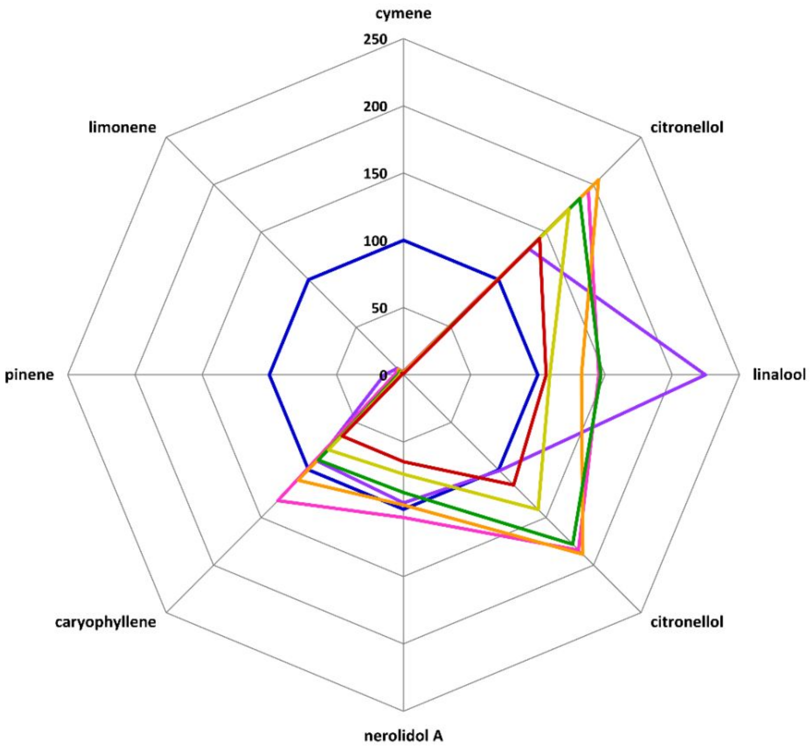

Figure S3b: Comparison of MS responses obtained using method 1 with ESCi source and various flow rates of make-up solvent, 0 (blue), 0.05 (purple), 0.1 (pink), 0.2 (orange), 0.3 (green), 0.4 (yellow), and 0.5 (red) mL/min.

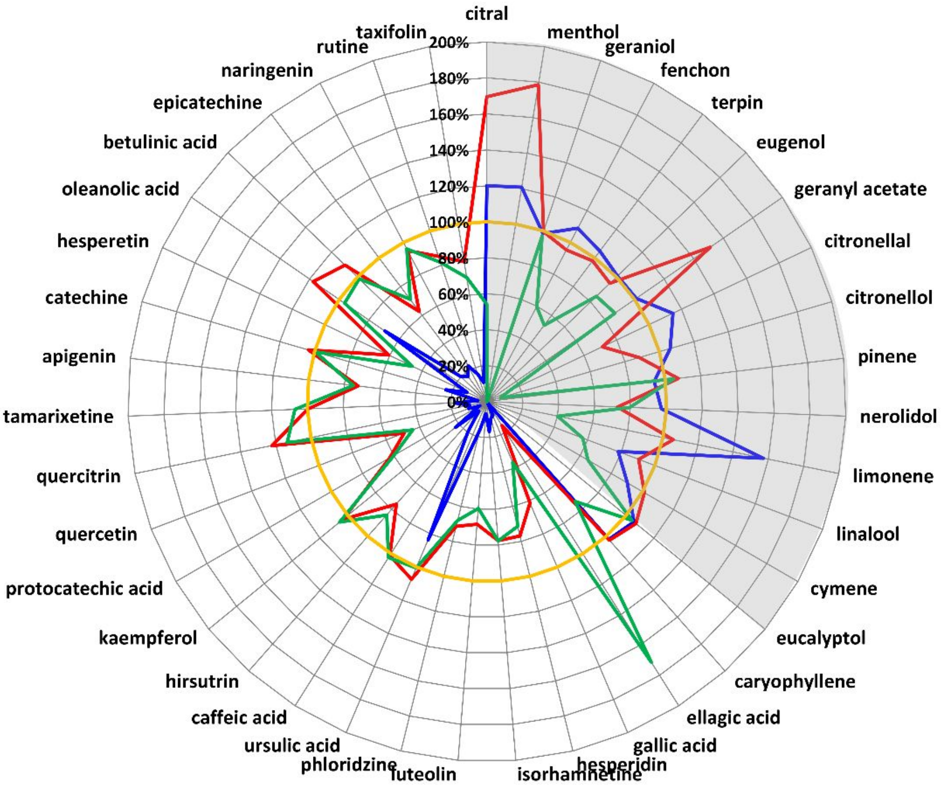

Figure S3c: Comparison of MS responses obtained using APCI source and various make-up solvents: 10 mmol/L ammonia in methanol (blue), 20 mmol/L ammonia in methanol (red), 50 mmol/L ammonia in methanol (green), 10 mmol/L ammonia + 10 mmol/L formic acid + 1% water in methanol (yellow). In gray – volatiles analyzed by method 1 with APCI source, in white – flavonoids analyzed by method 2 with APCI source.

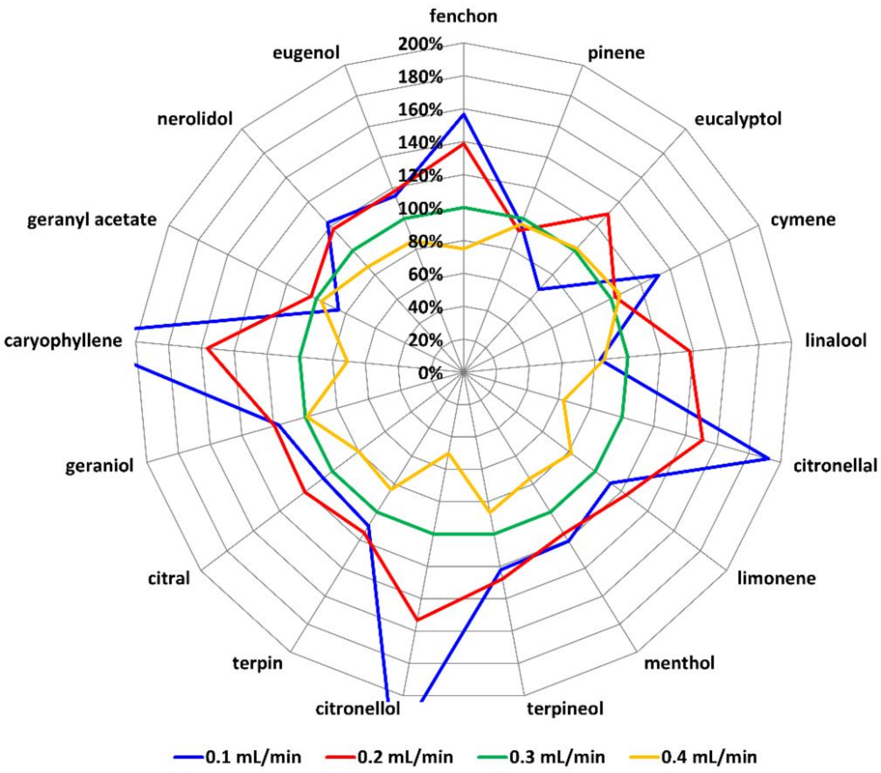

Figure S3d: Comparison of MS responses obtained using method 1 with APCI source and various flow rates of make-up solvent, i.e., 0.1 (blue), 0.2 (red) , 0.3 (green), and 0.4 (yellow) mL/min.

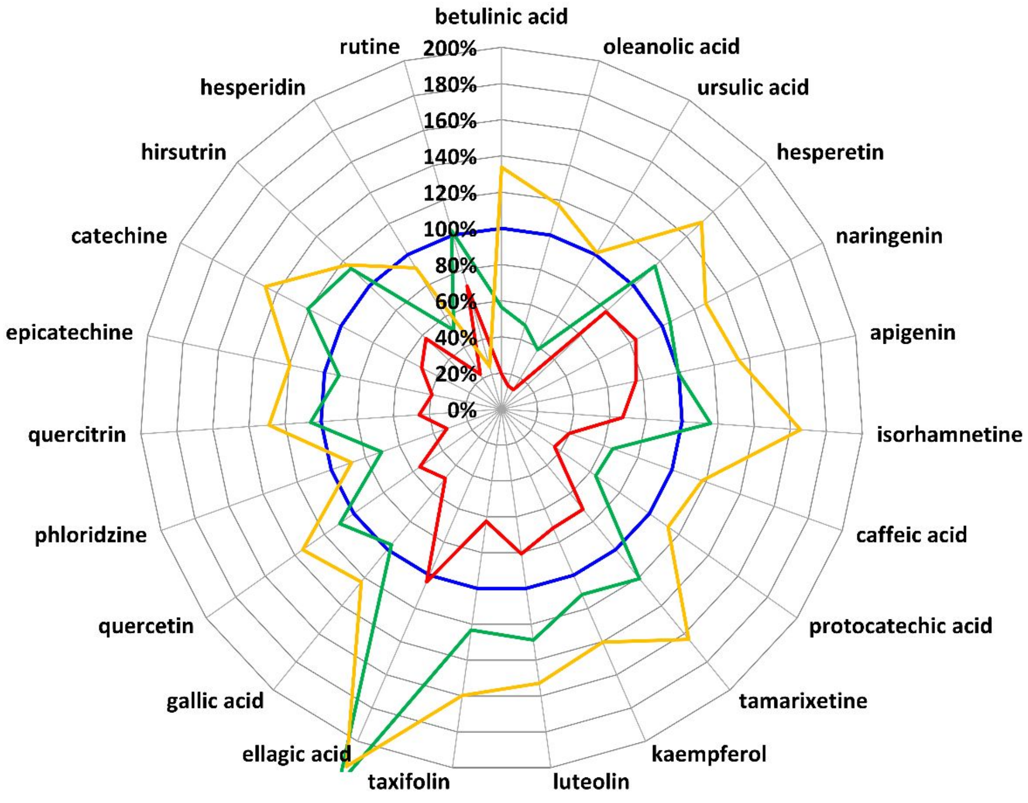

Figure S3e: Comparison of MS responses obtained using method 2 with UniSpray source using various make-up solvents, i.e., 10 mmol/L ammonia (yellow), 10 mmol/L formic acid (red), 1% water (green), and their combination (blue) in methanol.

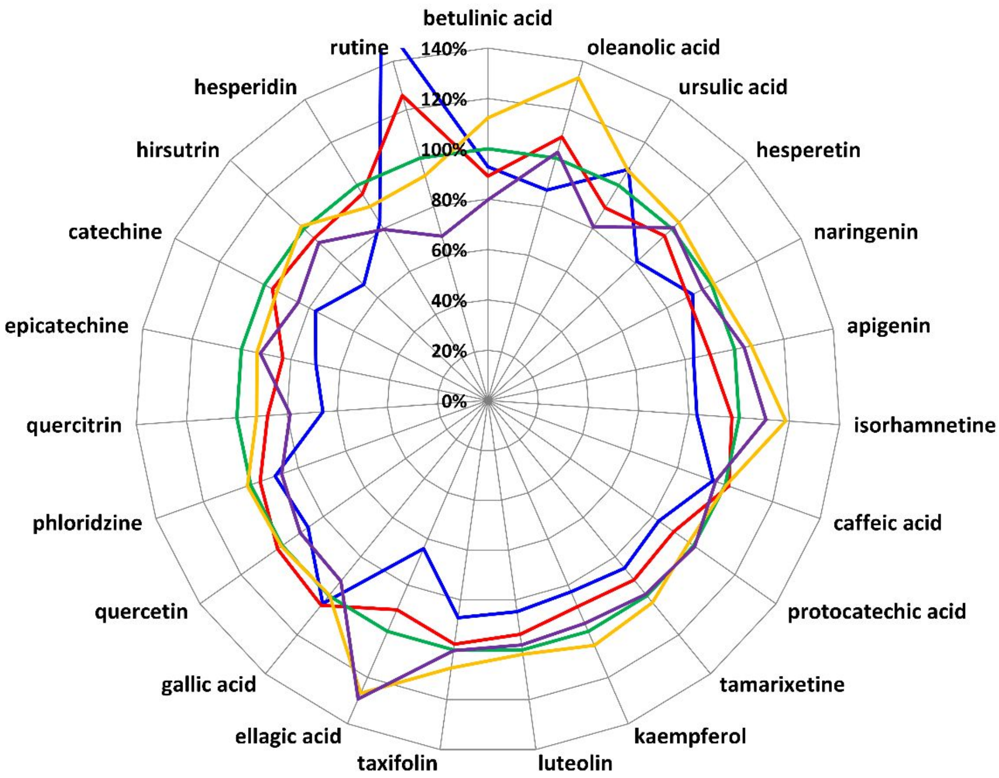

Figure S3f: Comparison of MS responses obtained using method 2 with UniSpray source and various flow rates of make-up solvent, i.e., 0.1 (blue), 0.2 (red) , 0.3 (green), 0.4 (yellow), and 0.5 (purple) mL/min.

S4. Design-of-experiments

Table S4: Contribution of tested parameters to the observed MS response using different ionization sources. 1- method 1, 2- method 2.

Factor contribution (%)	APCI - 1	APCI - 2	US - 1	US - 2	ESCi - 1	ESI - 2
corona current (μA)	14.7	21.0	-			-
desolvation gas flow rate (L/hr)	3.0	5.4	11.9	8.6	2.7	4.8
cone gas flow rate (L/hr)	35.0	4.0	14.4	29.2	5	29.8
nebulizer pressure (bar)	7.9	41.6	21.5	9.5	5	14.8
probe temperature (°C)	39.4	10.4	-			-
cone voltage (V)	-	17.6	-		25.2/10.8	17.7
impactor voltage (kV)	-	-	14.4	7.7		-
desolvation temperature (°C)	-	-	37.8	45.0	2	16.4
capillary voltage (eV)	-	-	-	-	26.1	16.5

S5. Lower limits of quantification

Table S5: Comparison of lower limits of quantification in ng/mL achieved using different ionization sources.

analyte	ESI/ESCi	US	APCI
**apigenin**	5.0	1.0	50.0
**betulinic acid**	5.0	7.0	2.0
**caffeic acid**	0.2	0.5	2.0
**catechin**	10.0	5.0	200.0
**ellagic acid**	20.0	0.5	500.0
**epicatechin**	10.0	5.0	200.0
**gallic acid**	7.0	20.0	700.0
**hesperetin**	2.0	1.0	50.0
**hesperidin**	50.0	10.0	200.0

**hirsutrin**	2.0	5.0	200.0
**isorhamnetin**	20.0	2.0	100.0
**kaempferol**	50.0	7.0	200.0
**luteolin**	7.0	1.0	100.0
**naringenin**	0.5	2.0	7.0
**oleanolic acid**	5.0	7.0	2.0
**phloridzine**	2.0	1.0	20.0
**protocatechuic acid**	0.7	5.0	100.0
**quercetin**	20.0	5.0	700.0
**quercitrin**	5.0	5.0	200.0
**rutin**	50.0	10.0	200.0
**tamarixetin**	7.0	1.0	500.0
**taxifolin**	10.0	2.0	500.0
**ursulic acid**	5.0	7.0	2.0
**eucalyptol**	20.0	10.0	1.0
**caryophyllene**	1.0	1.0	1.0
**citral**	5.0	5.0	1.0
**citronellal**	0.5	1.0	0.5
**citronellol**	5.0	5.0	0.5
**eugenol**	5.0	5.0	1.0
**fenchon**	2.0	10.0	1.0
**geraniol**	2.0	5.0	0.1
**geranyl acetate**	5.0	5.0	0.5
**limonene**	100.0	>1000	1.0
**linalool**	10.0	50.0	1.0
**menthol**	10.0	50.0	1.0
**nerolidol**	0.1	0.1	0.1
**p-cymene**	100.0	>1000	1.0
**t-terpin**	20.0	50.0	1.0
**α-pinene**	100.0	>1000	1.0
**α-terpineol**	100.0	100.0	1.0

S6. Comparison of different SFE extracts

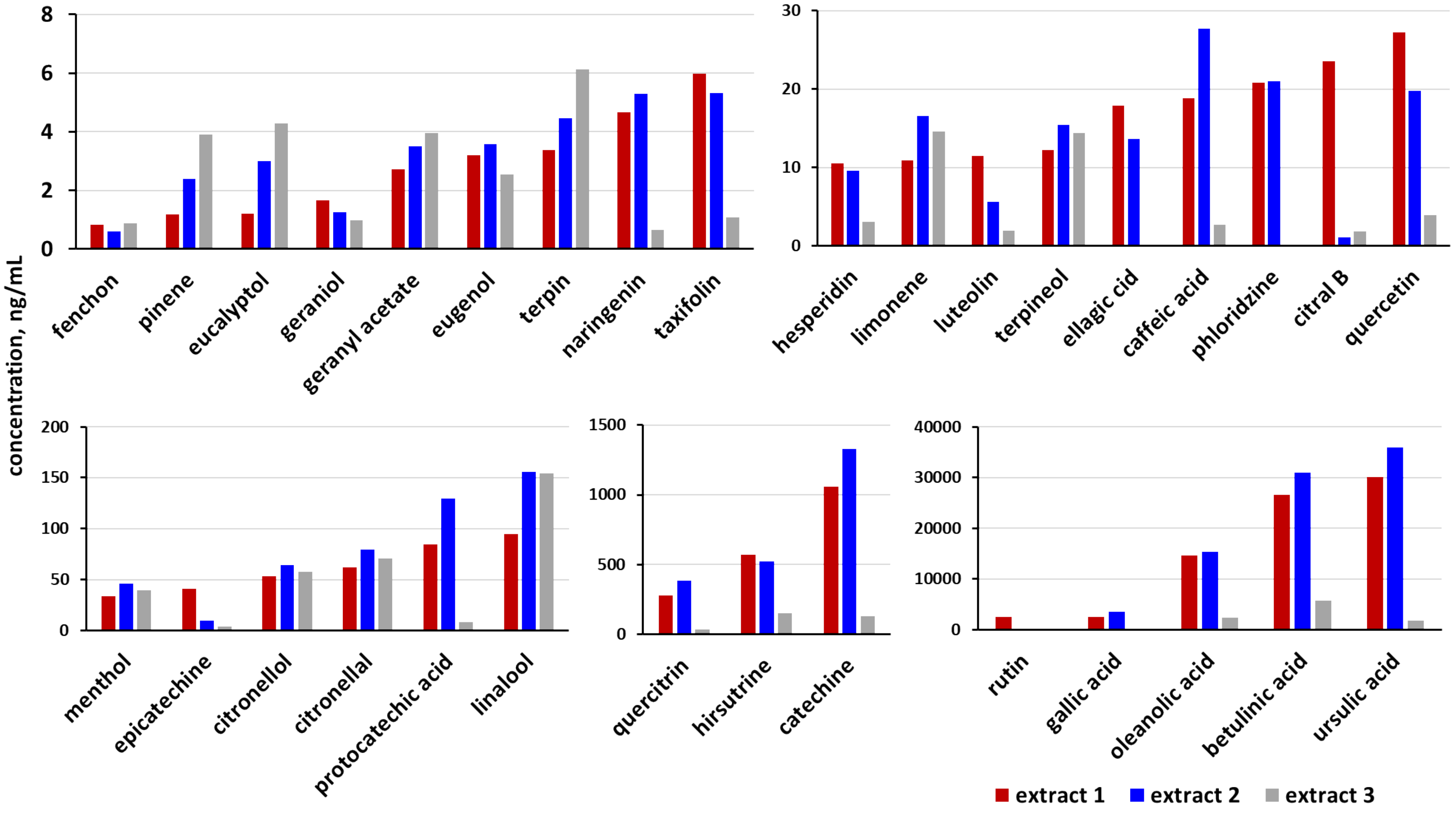

Figure S6: Comparison of concentrations of target analytes observed in three *Eucalyptus sp*. extracts obtained using three different SFE conditions. Extract 1: CO_2/EtOH 50/50, temperature 50 °C, pressure 16 MPa. Extract 2: neat CO_2, temperature 50 °C, pressure 30 MPa. Extract 3: neat CO_2, temperature 50 °C, pressure 16 MPa.
